# Diversification of African Tree Legumes in Miombo–Mopane Woodlands

**DOI:** 10.3390/plants8060182

**Published:** 2019-06-20

**Authors:** Ivete Maquia, Silvia Catarino, Ana R. Pena, Denise R.A. Brito, Natasha. S. Ribeiro, Maria M. Romeiras, Ana I. Ribeiro-Barros

**Affiliations:** 1 Linking Landscape, Environment, Agriculture and Food (LEAF), Instituto Superior de Agronomia (ISA), Universidade de Lisboa, Tapada da Ajuda, 1349-017 Lisbon, Portugal; 26415@novasbe.pt (I.M.); scatarino@isa.ulisboa.pt (S.C.); mmromeiras@isa.ulisboa.pt (M.M.R.); 2 Biotechnology Center, Eduardo Mondlane University, Av. de Moçambique Km 1.5, Maputo 1109, Mozambique; nisebrito@gmail.com; 3 Forest Research Center (CEF), Instituto Superior de Agronomia (ISA), Universidade de Lisboa, Tapada da Ajuda, 1349-017 Lisboa, Portugal; 4 Centre for Ecology, Evolution and Environmental Changes (cE3c), Faculdade de Ciências, Universidade de Lisboa, 1749-016 Lisbon, Portugal; ritah_catitah@hotmail.com; 5 Faculty of Agronomy and Forest Engineering, Eduardo Mondlane University, Campus1, P.O. Box 257, Maputo 1102, Mozambique; nribeiro@uem.mz; 6 Unidade de Geobiociências, Geoengenharias e Geotecnologias (GeoBioTec), Faculdade de Ciências e Tecnologia, Universidade NOVA de Lisboa, 2829-516 Monte de Caparica, Portugal

**Keywords:** diversity, Miombo, Mopane, tree legumes, Zambezian phytoregion

## Abstract

The southern African Miombo and Mopane ecoregions constitute a unique repository of plant diversity whose diversification and evolutionary history is still understudied. In this work, we assessed the diversity, distribution, and conservation status of Miombo and Mopane tree legumes within the Zambezian phytoregion. Data were retrieved from several plant and gene databases and phylogenetic analyses were performed based on genetic barcodes. Seventy-eight species (74 from Miombo and 23 from Mopane, 19 common to both ecoregions) have been scored. Species diversity was high within both ecoregions, but information about the actual conservation status is scarce and available only for ca. 15% of the species. Results of phylogenetic analyses were consistent with current legume classification but did not allow us to draw any conclusion regarding the evolutionary history of Miombo and Mopane tree legumes. Future studies are proposed to dissect the diversity and structure of key species in order to consolidate the network of conservation areas.

## 1. Introduction

Tropical dry forests and woodlands constitute a large portion of the world’s vegetation, covering one-sixth of the earth’s surface and more than half of the African continent [1,2]. Among them, the Miombo–Mopane woodlands are the most predominant type of vegetation in Southern Africa, and together with Amazonia, Congo, New Guinea and the North American deserts, are considered wilderness areas of global conservation significance [3]. The woodlands play a crucial role in formal and informal economies, supporting the livelihoods of millions of rural and urban people, by providing important resources such as timber, food, medicines, biofertilizers, housing and energy [4,5,6,7,8]. The Miombo and Mopane woodlands also play an important role in the ecosystem dynamics, particularly with respect to biodiversity, water, carbon and energy balance [9,10,11,12,13].

The plant diversity of these ecosystems comprises a wealthy repository of biodiversity, with a high proportion of native species, which makes it biologically unique [14,15]. According to Olson et al. [16], five sub-regions have been delineated through the Miombo woodlands (i.e., Angolan Miombo woodlands, Central Zambezian Miombo woodlands, Zambezian Baikiaea woodlands, Eastern Miombo woodlands and Southern Miombo woodlands) that cover about 3,000,000 km^2^ across the Zambezian region of Angola, Democratic Republic of Congo, Malawi, Mozambique, Tanzania, Zambia and Zimbabwe [17,18]. The Mopane woodlands represent the second most significant type of vegetation in the Zambezian phytoregion, covering approximately 600,000 km^2^. This region includes two sub-areas (i.e., Zambezian and Mopane woodlands, and Angolan Mopane woodlands), and is distributed over northern Namibia, southern Angola, Zimbabwe, Botswana, Zambia, Malawi, southern Mozambique and northern South Africa [17,19].

The Miombo and Mopane woodlands are dominated by species belonging to the Leguminosae [2], which is considered the second most economically important plant family [20,21,22,23]. This family includes over 19,500 species spanning about 770 genera and six subfamilies, namely Caesalpinioideae, Cercidoideae, Detarioideae, Dialioideae, Duparquetioideae and Papilionoideae, many of which establish root-nodule symbiosis with N_2_ fixing rhizobia bacteria [23]. The Miombo woodlands are dominated by trees of the genera *Brachystegia*, *Julbernardia* and *Isoberlinia*, while the Mopane woodlands are dominated by *Colosphosperum mopane* (Benth.) Leonard [9,17,24]. Most of these trees are under severe ecological pressure, due to logging and charcoal production [25,26], as well as fires related to animal, human and climate factors [6,10,11,13], which have contributed to the massive degradation of these woodlands and raised the need for their conservation [4,24,27,28].

**Figure 1:**
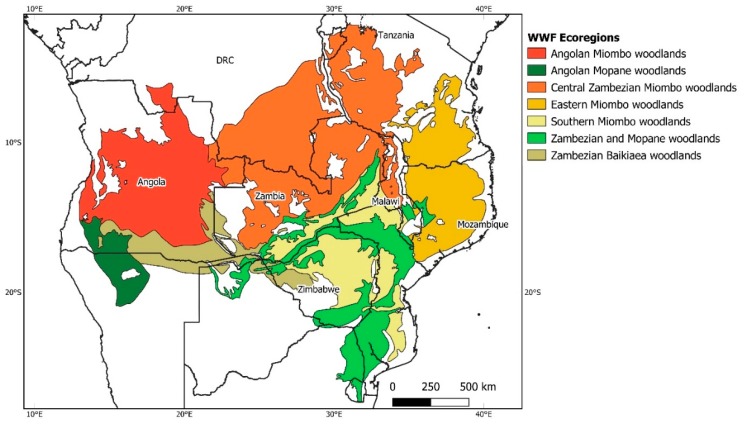
Geographical distribution of the Miombo–Mopane woodlands in the Zambezian phytoregion.

African forests are among the most understudied regions in the world with respect to phylogenetic diversity [29]. Characterization of a region’s phylogenetic diversity, which is based on the evolutionary relationships between species from a given geographical area, can be a powerful tool to analyze the phylogenetic structure of natural communities and can assist the study of processes governing the assembly of species in ecological communities [30,31]. The identification of areas with more or less phylogenetic diversity based on species richness is another important element for conservation studies [32]. Although the African woodlands remain of enormous evolutionary interest, assessing species diversification within Miombo and Mopane plant communities across West and East African woodlands is still understudied. Since the dominant plant lineages in Miombo and Mopane are tree legumes, this provides an excellent case study to understand the diversity and the evolutionary history of these two predominant types of vegetation in Southern Africa. In this context, the objectives of this study were: (i) to characterize the diversity and distribution of Miombo and Mopane tree legumes within the Zambezian phytoregion (Figure 1); (ii) to provide a unique view of their phylogenetic diversity and how these lineages have evolved across West and East African woodlands; and (iii) to provide new data to assist the implementation of conservation strategies and the sustainable management of the Mopane and Miombo woodlands.

## 2. Results

### 2.1. Tree Legumes Diversity and Conservation Status

Our study categorized 78 Leguminosae trees, of which 74 were representative of the Miombo woodlands and 23 of the Mopane woodlands (19 species common to both habitats) (Table 1). Overall, the largest number of species was found in Zambia, with 71 out of the 78 species (91%), while the Democratic Republic of Congo (DRC) had the lowest number (51 species or 65%) (Table 1). Five of the six recognized subfamilies were found in the Miombo woodlands: Caesalpinioideae, the most frequent (27 species), followed by Papilionoideae (22 species), Detarioideae (21 species), and Cercidoideae and Dialioideae with only three and one species, respectively. Among the 34 genera, *Brachystegia* and *Acacia* were the most diverse with 12 and 11 species, respectively. The number of species per country ranged from 50 in the DRC to 67 in Zambia. The Mopane woodlands harbored four subfamilies of legume trees, 14 species belonging to the Caesalpinioideae subfamily, followed by Papilionoideae with six species. Only two species from Detarioideae and one from Cercidoideae were retrieved. The number of genera was lower in Mopane than in Miombo (15 versus 34). The highest number of species was recorded in Zimbabwe (23) and the lowest in the DRC (11).

**Figure 2:**
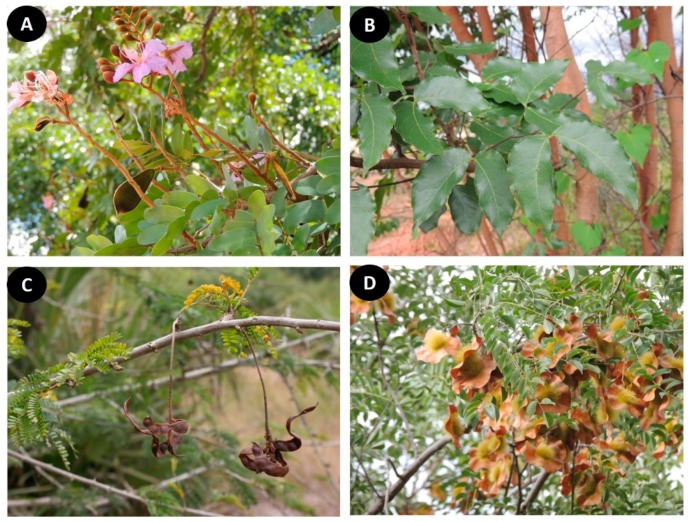
*Leguminosae* species from Miombo and Mopane woodlands and their conservation status according to the IUCN Red List. (**A**) *Baikiaea plurijuga* occurs in Miombo and Mopane woodlands and is classified as near threatened; (**B**) *Brachystegia bakeriana* occurs in Miombo and is classified as vulnerable; (**C**) *Dichrostachys cinerea* occurs in both ecoregions and is classified as least concern; (**D**) *Pterocarpus angolensis* occurs in Miombo woodlands and is classified as near threatened.

Information on the conservation status of the Miombo and Mopane trees is still scarce as most of the species (66 species or 85%) have not yet been assessed globally, according to the categories and criteria of the International Union for Conservation of Nature (IUCN) Red List (Table 1). Therefore, only 12 species could be evaluated, of which seven were classified with least concern (*Acacia nilotica* (L.) Willd. ex Delile; *Baphia massaiensis* Taub.; *Brachystegia puberula* Burtt Davy & Hutch.; *Dichrostachys cinerea* (L.) Wight & Arn.; *Pterocarpus brenanii* Barbosa & Torre; *Pterocarpus lucens* Guill. & Perr.; *Tamarindus indica* L.), three as near threatened (*Dalbergia melanoxylon* Guill. & Perr.; *Pterocarpus angolensis* DC.; *Baikiaea plurijuga* Harms), and two as vulnerable species (*Isoberlinia scheffleri* (Harms) Greenway; *Brachystegia bakeriana* Burtt Davy & Hutch) (Figure 2).

### 2.2. Molecular Phylogenetic Analysis

As DNA sequences were not available for all the selected taxa, the analyzed set was reduced to a total of 67 species from the original set of 78 (Table 2). A total of eight datasets were created: two for each single locus (internal transcribed spacer (ITS), matK and rbcL), and two combined (ITS + matK + rbcL). The ITS matrix had the lower number of taxa and haplotype diversity (n = 38, Hd = 0.9986), while rbcL had the highest of the three (n = 53, Hd = 1.0000), followed by matK (n = 69, Hd = 0.9987). The combined set of ITS+matK+rbcL had a total of 70 sequences, with a single shared haplotype (Hd = 0.9996). A summary of the processed molecular data of this study is available in Table S1 of the Supplementary Materials.

For each data matrix, two independent phylogenies were constructed, one using maximum likelihood (ML), and another using Bayesian inference (BI). In both methods, tree rooting was performed using *Polygalaceae* species as outgroups, namely *Monnina xalapensis*, *Rhinotropis acanthoclada* and *Xanthophyllum hainanense*. Overall, the BI phylogenies achieved higher support values, but were in turn less resolved, with more polytomies than ML trees. Topologies were highly similar within the same paired datasets, and to a lesser extent between locus, showing similar clustering patterns between species. 

**Figure 3:**
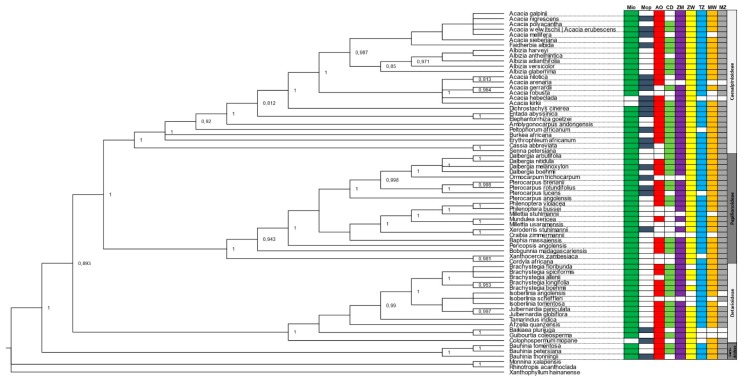
Phylogenetic tree of the Leguminosae tree species from Miombo and Mopane ecosystems. The phylogeny was constructed with three selected genes (ITS + matK + rbcL) using the Bayesian inference (BI) method. Acronyms for the countries/regions: Mio—Miombo; Mop—Mopane; AO—Angola; DRC—Democratic Republic of the Congo; ZM—Zambia; ZW—Zimbabwe; TZ—United Republic of Tanzania; MW—Malawi; MZ—Mozambique.

**Figure 4:**
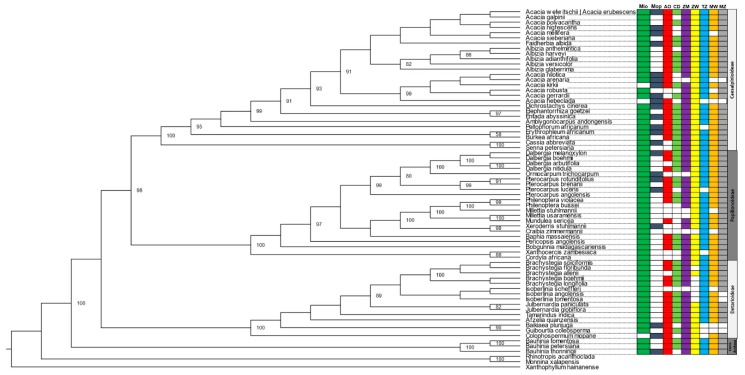
Phylogenetic tree of the Leguminosae tree species from Miombo and Mopane ecosystems. The phylogeny was constructed with three selected genes (ITS + matK + rbcL) and the maximum likelihood (ML) method. Acronyms for the countries/regions: Mio—Miombo; Mop—Mopane; AO—Angola; DRC—Democratic Republic of the Congo; ZM—Zambia; ZW—Zimbabwe; TZ—United Republic of Tanzania; MW—Malawi; MZ—Mozambique.

The phylogenetic trees obtained using the matK matrix provided the best resolution and support from the singular gene analysis, showing a clear split between subfamilies and clustering between closely resembling species (Figure S1). ITS and rbcL phylogenies were generally less supported in both ML and BI analysis (Figures S2 and S3), and some taxa were also poorly positioned (i.e., found outside of the subfamily clade). The concatenated set of the three selected genes (ITS + matK + rbcL) achieved the best overall result both using BI (Figure 3) and ML (Figure 4), with a lower number of unresolved nodes, as well as higher values of support, following the clustering pattern obtained by The Legume Phylogeny Working Group [23].

The distribution of species among ecoregions (Miombo and Mopane) was as predictable and in general evenly distributed across the seven countries. The smaller subfamily, Cercidoideae, was well represented in Miombo (3 out of 3 species), holding a single taxon (*Bauhinia thonningii*), which was found in Mopane as well. The Detarioideae subfamily included 15 species, one common to both Miombo and Mopane (*Baikiaea plurijuga*), one exclusively from Mopane (*Colophospermum mopane*) and 13 exclusively from Miombo (*Brachystegia* spp., *Isoberlinea* spp., *Julbernardia* spp., *Tamarindus indica* and *Guibourtia coleosperma*). One of these species, *I. scheffleri*, was restricted to Tanzania and Mozambique, while *B. plurijuga* and *G. coleosperma* were not present in Tanzania, Malawi, Mozambique and the DRC. The 21 species belonging to the Papilionoideae were all present in Miombo, five of them (*Dalbergia melanoxylon*, *Ormocarpum trichocarpum*, *Pterocarpus rotundifolius*, *Pterocarpus lucens*, and *Xeroderris stuhlmannii*) being also present in Mopane. Three species, *O. trichocarpum*, *Millettia stuhlmannii* and *M. usaramensis* were present only in the eastern countries. The largest subfamily Caesalpinioideae, included 25 species, mostly *Acacia* spp. and *Albizia* spp. homogeneously distributed by country and ecoregion (Figure 3 and Figure 4). 

## 3. Discussion

As expected, the Miombo woodlands presented a higher diversity of species (74 overall taxa, 55 Miombo-exclusive) than Mopane (23 taxa, 4 Mopane-exclusive) [17,33]. This is likely related to the larger area and therefore to a rather diverse edaphic (e.g., drainage, soil depth and texture) and climate (warm to hot climate, 710 to 1365 mm mean annual precipitation and 18 to 23 °C mean annual temperature) conditions in the Miombo ecoregion [9,34]. Additionally, Mopane is usually characterized by clayed soils [33] with a discontinuous tree cover and a continuous C4 grass layer [35,36]. Thus, environmental determinants might have underlined a slight deviation on the evolutionary history of Miombo and Mopane tree legumes [37]. Also expected was the exclusive presence of the typically dominant genera of Miombo (*Brachystegia*, *Isoberlinia* and *Julbernardia*) and Mopane (*Colophospermum mopane*) in either ecosystem [17,19,24].

When comparing the results with the combined areas of Miombo and Mopane woodlands there was not a logical correlation between area size and species number in some countries. This was particularly evident in Angola, which holds the largest woodland area of Miombo and Mopane but houses only 62 out of the 78 species. Other large countries such as Zambia and Mozambique however, did not follow this trend, as the identified species were well represented. These results could be explained by three hypotheses: i) the origin point of dispersal for the Leguminosae family was Zambia and/or Zimbabwe with a more recent expansion to Angola, compared to the other neighboring countries; ii) the knowledge on species diversity in Angola is incipient; or iii) a combination of the two. Among the seven countries included in this study, the highest species diversity was found in Zambia (i.e., 71 out of the 78 scored species, and 16 out of 17 typical Miombo species), likely related to the fact that Zambia is the center of endemism for *Brachystegia* [9]. Except for Zambia, the number of taxa was nearly the same for countries with dry Miombo (Malawi, Mozambique and Zimbabwe) and wet Miombo (Angola, DRC, Tanzania and Malawi). However, it is important to consider that the scored diversity in this study does not reflect species abundance and frequency, which could explain this similarity despite the fact that wet Miombo is often a floristically richer region [9,38].

The rarity of a certain species or ecosystem is frequently the first and most important feature when deciding its need of protection, as is the higher risk of extinction and loss of possible unique lineages [39,40,41]. This feature is particularly significant when information on the threat status of a species is insufficient [42] as is the case of Miombo and Mopane tree legumes, whose conservation status is available for a minority of species (Table 1) [43]. Such a trend was also found for other tree lineages endemic from Africa such as the nitrogen-fixing actinorhizal trees and shrubs [44]. This issue is of upmost importance within the context of the Bonn Challenge under which many countries have pledged to restore millions of degraded and deforested woodlands and forests [45]. Thus, more efforts are needed to investigate the vegetation dynamics, anthropogenic and environmental drivers as well as the different conservation management strategies across Miombo and Mopane countries. Examples of such efforts include the recent work of, (i) Chiteculo and Surovy [46] and Chiteculo et al. [47], that characterized the vegetation composition and structure and deforestation patterns of the Miombo woodlands in the Huambo province, Angola, respectively; (ii) Ribeiro et al. [6] that conducted a 12-year analysis of the spatio-temporal patterns of fire to refine the fire management strategy in one of the most pristine areas of Miombo, the Niassa National Reserve, Mozambique; (iii) Mugasha et al. [48] that provided a pioneer study on modeling tree growth in the Miombo woodlands from Tanzania based on long-term monitoring data; and (iii) Chidumayo [49] that performed a long-term study (1982–2018) across the Miombo woodlands in Zambia to investigate the woodland drivers and contribute to the design of management plans.

The use of phylogenetic diversity as an effective complementary mean of conservation has often been the object of debate. Since the proposal of this metric by Vane-Wright [50], several studies have been able to test and evaluate this hypothesis with various results and opinions [51,52]. Regarding molecular data, the results obtained in this study corroborate previous molecular findings showing that the use of taxonomic barcodes or DNA barcoding have great applicability on the identification, phylogeny reconstruction and evolutionary analysis for forest dwelling flora [53,54,55], in our case tree legumes from the Miombo and Mopane ecoregions. The results are in line with a recent study by The Legume Phylogeny Working Group [23], clustering together four out of the six new subfamilies: Caesalpinioideae, Cercidoideae, Detarioideae and Papilionoideae (Figure 3 and Figure 4). 

Functional diversity is another useful parameter to assess phylogenetic diversity and the evolutionary potential (i.e., the ability of a species to adapt to environmental changes) [56,57,58,59]. With few exceptions, the distribution of the 67 lineages and subfamilies across the seven countries was quite uniform and therefore, not informative regarding their evolutionary history across the Mopane and Miombo axis. This was particularly the case of the typically dominant genera of Miombo (i.e., *Brachystegia*, *Isoberlinia* and *Julbernardia*) and Mopane (*C. mopane*). Thus, further studies are needed in order to assess the genetic diversity and population structure of key species from Miombo and Mopane. Such complementary studies will be essential to provide better and more well-founded areas of protection for the Miombo and Mopane woodlands. In some countries where protected areas are still scarce, such as Angola, this might support the establishment of a network of protected areas spanning different sub-regions. 

In conclusion, the Miombo and Mopane woodlands hold a differential phylogenetic diversity: the Miombo covers a larger area and holds a higher number of legume species; while the Mopane spans a smaller land mass that houses several unique and rare lineages. Both ecoregions hold a high value of biodiversity, even with a somewhat dissimilar composition, and as such future studies should take in account their exclusive characteristics alongside their shared ones when proposing new conserved species and areas.

## 4. Materials and Methods 

### 4.1. Study Area and Spatial Analyses

The study area included the Mopane and Miombo ecoregions from the Zambezian phytoregion (i.e., Angola, Democratic Republic of Congo (DRC), Zambia, Zimbabwe, Tanzania, Malawi, and Mozambique) (Figure 1). Only the southern provinces of DRC (i.e., Tanganyika, Haut-Lomami, Lualaba, and Haut-Katanga (former province of Katanga, extinguished in 2009)), were included in this study, as only these four provinces are part of the Zambezian phytoregion [60]. The map of the terrestrial World Wildlife Fund (WWF) ecoregions [61] and the five Miombo and two Mopane sub-regions were analyzed and overlapped with the digital maps of the Zambezian region.

### 4.2. Database of Legume Trees 

A list of the native tree legume species (Leguminosae family) from the Mopane and Miombo woodlands was created through an extensive research in scientific publications [6,12,17,33,62] and references therein and online databases, such as the African Plant Database [63], Plants of the World Online [64], Flora of Mozambique [65], Flora of Zambia [66], Flora of Malawi [67] and Flora of Zimbabwe [68]. Scientific names were updated according to The Plant List [69], while subfamilies were compiled using the organization defined by the The Legume Phylogeny Working Group (LPWG) [23]. The main distribution in the Zambezian phytoregion, species’ habit, conservation status and molecular data were also searched and compiled. Distribution data was attained in several bibliographic sources [70,71,72,73,74] and online databases, namely the Global Biodiversity Information Facility (GBIF) platform [75], and the African Plant Database [63]. The conservation status of each species was consulted in the International Union for Conservation of Nature—Red List [40]. 

### 4.3. Sequence Data and Phylogenetic Analysis

To perform the molecular analysis, three markers were selected: two chloroplast genes often used in plant barcoding, rbcL and matK [76,77]; and the internal transcribed spacer (ITS) region, which has been shown to be a useful complementary barcode [78].

The software Geneious Prime 2019.0.3 [79] was used to retrieve the selected DNA sequences from the National Center for Biotechnology Information (NCBI) GenBank database, and each locus dataset was subsequently aligned using the multiple sequence alignment tool MAFFT [80], available online [81]. The haplotype diversity of the data was verified using DNASP6 v.6.12.01 [82], and then processed with trimAl v.1.3 [83], through the Phylemon2 framework [84], to remove poorly aligned regions and improve the quality of the alignments. A concatenated set using the three loci was created using Concatenator [85] to assess the collective phylogeny of the selected taxa. PartitionFinder2 v.2.1.1 [86,87,88] was used to find the adequate partitioning and models of evolution for the four groups (three single and one combined), using the corrected Akaike Information Criterion (AICc) as the model, and partitioning by gene and codon position. 

Phylogeny reconstructions were made using two different methodologies for the individual and combined locus datasets, namely maximum likelihood (ML) and Bayesian inference (BI). In both cases, species from the Polygalaceae family, a sister group of Leguminoseae, were selected as outgroups (*Monnina xalapensis*, *Rhinotropis acanthoclada* and *Xanthophyllum hainanense*, the latter functioning as the most exterior outgroup) [89]. ML analyses were performed using RAxML v.8.2.10 [90], through RaxmlGUI v.1.5b [91], using a ML + rapid bootstrap search and an autoMRE bootstrap. BI analyses were made using MrBayes v.3.2.6 [92], with 1.5 × 107 generations and a sample every 100 steps, with default chains and temperature. Convergence on all parameters was verified using Tracer v.1.6 [93] across all runs. The BI analyses were performed using the Cipres Gateway services [94]. Finally, we summarized, annotated and later exported the resulting phylogenetic trees using FigTree v.1.4.3 [95].

## Figures and Tables

**Table 1:** Leguminosae tree species from Miombo and Mopane ecosystems: main habitat, distribution in the Zambezian area, conservation status (International Union for Conservation of Nature - IUCN) and habit.

Taxon	Subfamily	Habitat	Distribution ^1^							Conservation Status ^2^	Habit
			AO	DRC	ZM	ZW	TZ	MW	MZ		
*Acacia arenaria* Schinz	Caesalpinioideae	Miombo and Mopane	X			X	X			NE	Shrub or small tree 2–9 m
*Acacia erubescens* Welw. ex Oliv.	Caesalpinioideae	Miombo and Mopane	X	X	X	X	X	X	X	NE	Shrub or tree 2–10 m
*Acacia galpinii* Burtt Davy	Caesalpinioideae	Miombo	X		X	X	X	X	X	NE	Large tree 8–36 m
*Acacia gerrardii* Benth.	Caesalpinioideae	Miombo and Mopane		X	X	X	X	X	X	NE	Shrub or tree 5–15 m
*Acacia hebeclada* DC.	Caesalpinioideae	Mopane	X		X	X				NE	Shrub or small tree to 3 m
*Acacia kirkii* Oliv.	Caesalpinioideae	Mopane	X	X	X	X	X	X	X	NE	Tree 2.5–18 m
*Acacia mellifera* (Vahl) Benth.	Caesalpinioideae	Miombo and Mopane	X		X	X	X		X	NE	Tree 4–9
*Acacia nigrescens* Oliv.	Caesalpinioideae	Miombo and Mopane	X		X	X	X	X	X	NE	Tree 4–30 m
*Acacia nilotica* (L.) Willd. ex Delile	Caesalpinioideae	Miombo and Mopane	X		X	X	X	X	X	LC	Tree 3–15 m
*Acacia polyacantha*	Caesalpinioideae	Miombo	X	X	X	X	X	X	X	NE	Large tree 3.5–20 m
*Acacia robusta* Burch.	Caesalpinioideae	Miombo			X	X	X		X	NE	Tree 5-30 m
*Acacia sieberiana* DC.	Caesalpinioideae	Miombo	X	X	X	X	X	X	X	NE	Tree 3–25 m
*Acacia welwitschii* Oliv.	Caesalpinioideae	Miombo	X			X			X	NE	Large tree 3–20 m
*Afzelia quanzensis* Welw.	Detarioideae	Miombo	X	X	X	X	X	X	X	NE	Shrub or tree 1.5–35 m
*Albizia adianthifolia* (Schum.) W.Wight	Caesalpinioideae	Miombo	X	X	X	X	X	X	X	NE	Tree 2.5–40 m
*Albizia anthelmintica* Brongn.	Caesalpinioideae	Miombo	X		X	X	X	X	X	NE	Shrub or tree 2–12 m
*Albizia antunesiana* Harms	Caesalpinioideae	Miombo	X	X	X	X	X	X	X	NE	Tree 6–18 m
*Albizia glaberrima* (Schum. & Thonn.) Benth.	Caesalpinioideae	Miombo	X	X	X	X	X	X	X	NE	Shrub or tree 9–25 m
*Albizia harveyi* E.Fourn.	Caesalpinioideae	Miombo	X	X	X	X	X	X	X	NE	Tree 1.5–20 m
*Albizia versicolor* Oliv.	Caesalpinioideae	Miombo	X	X	X	X	X	X	X	NE	Tree 3–20 m
*Amblygonocarpus andongensis* (Oliv.) Exell & Torre	Caesalpinioideae	Miombo	X	X	X	X	X	X	X	NE	Tree 6–25 m
*Baikiaea plurijuga* Harms	Detarioideae	Miombo and Mopane	X		X	X				LR/NT	Tree 6–25 m
*Baphia bequaertii* De Wild.	Papilionoideae	Miombo	X	X	X					NE	Shrub or tree 3–10 m
*Baphia massaiensis* Taub.	Papilionoideae	Miombo	X	X	X	X	X	X	X	LC	Shrub or tree to 8–10 m
*Bauhinia petersiana* Bolle	Cercidoideae	Miombo	X	X	X	X	X	X	X	NE	Shrub or tree 3–10 m
*Bauhinia thonningii* Schum.	Cercidoideae	Miombo and Mopane	X		X	X	X	X	X	NE	Shrub or tree to 2–20 m
*Bauhinia tomentosa* L.	Cercidoideae	Miombo	X	X	X	X	X	X	X	NE	Shrub or tree 1–8 m
*Bobgunnia madagascariensis* (Desv.) J.H.Kirkbr. & Wiersema	Papilionoideae	Miombo	X	X	X	X	X	X	X	NE	Shrub or tree 2–15 m
*Brachystegia allenii* Hutch. & Burtt Davy	Detarioideae	Miombo		X	X	X	X	X	X	NE	Tree 3–20 m
*Brachystegia bakeriana* Burtt Davy & Hutch.	Detarioideae	Miombo	X		X	X				VU B1+2c	Shrub or tree to 10 m
*Brachystegia boehmii* Taub	Detarioideae	Miombo	X	X	X	X	X	X	X	NE	Tree 2.5–25 m
*Brachystegia floribunda* Benth.	Detarioideae	Miombo	X	X	X		X	X	X	NE	Tree 4–15 m
*Brachystegia gossweileri* Burtt Davy & Hutch.	Detarioideae	Miombo	X	X	X					NE	Tree 6–24 m
*Brachystegia longifolia* Benth.	Detarioideae	Miombo	X	X	X		X	X	X	NE	Tree 2–30 m
*Brachystegia puberula* Burtt Davy & Hutch.	Detarioideae	Miombo	X		X		X			LC	Tree 6–12 m
*Brachystegia spiciformis* Benth	Detarioideae	Miombo	X	X	X	X	X	X	X	NE	Tree 5–40 m
*Brachystegia tamarindoides* Benth.	Detarioideae	Miombo	X		X		X	X	X	NE	Tree 4–30 m
*Brachystegia taxifolia* Harms	Detarioideae	Miombo		X	X		X	X		NE	Shrub or tree 2–16 m
*Brachystegia utilis* Hutch. & Burtt Davy	Detarioideae	Miombo	X	X	X	X	X	X	X	NE	Tree 6–20 m
*Brachystegia wangermeeana* De Wild.	Detarioideae	Miombo	X	X	X		X	X		NE	Tree 1.5–20 m
*Burkea africana* Hook.	Caesalpinioideae	Miombo	X	X	X	X	X	X	X	NE	Tree 4–20 m
*Cassia abbreviata* Oliv.	Caesalpinioideae	Miombo and Mopane		X	X	X	X	X	X	NE	Shrub or tree 3–15 m
*Colophospermum mopane* (Benth.) Leonard	Detarioideae	Mopane	X		X	X		X	X	NE	Tree 4–18 m tall
*Cordyla africana* Lour.	Papilionoideae	Miombo			X	X	X	X	X	NE	Tree 9–40 m
*Craibia zimmermannii* (Harms) Dunn	Papilionoideae	Miombo					X		X	NE	Tree 4–5 m
*Dalbergia arbutifolia* Baker	Papilionoideae	Miombo		X	X	X	X	X	X	NE	Shrub or tree 3–18 m
*Dalbergia boehmii* Taub.	Papilionoideae	Miombo	X	X	X	X	X	X	X	NE	Shrub or tree 4.5–21 m
*Dalbergia melanoxylon* Guill. & Perr.	Papilionoideae	Miombo and Mopane	X	X	X	X	X	X	X	LR/NT	Spiny shrub or tree 1–30 m
*Dalbergia nitidula* Baker	Papilionoideae	Miombo	X	X	X	X	X	X	X	NE	Shrub or tree 2–12 m
*Dialium englerianum* Henriq.	Dialioideae	Miombo	X	X	X	X				NE	Tree 6–23 m
*Dichrostachys cinerea* (L.) Wight & Arn.	Caesalpinioideae	Miombo and Mopane	X	X	X	X	X	X	X	LC	Shrub or tree 1–12 m
*Elephantorrhiza goetzei* (Harms) Harms	Caesalpinioideae	Miombo	X	X	X	X	X	X	X	NE	Shrub or tree 1–7 m
*Entada abyssinica* Steud. ex A. Rich.	Caesalpinioideae	Miombo and Mopane	X	X	X	X	X	X	X	NE	Tree 2.7–15 m
*Erythrophleum africanum* (Benth.) Harms	Caesalpinioideae	Miombo and Mopane	X	X	X	X	X	X	X	NE	Tree 4–18 m
*Faidherbia albida* (Delile) A.Chev.	Caesalpinioideae	Miombo and Mopane	X	X	X	X	X	X	X	NE	Tree 6–30 m
*Guibourtia coleosperma* (Benth.) J. Léonard	Detarioideae	Miombo	X	X	X	X				NE	Tree 12–30 m
*Isoberlinia angolensis* (Benth.) Hoyle & Brenan	Detarioideae	Miombo	X	X	X		X	X		NE	Shrub or tree 1–20 m
*Isoberlinia scheffleri* (Harms) Greenway	Detarioideae	Miombo					X		X	VU B1+2b	Tree 30–46 m
*Isoberlinia tomentosa* (Harms) Craib & Stapf	Detarioideae	Miombo	X	X	X		X	X		NE	Tree 3–18 m
*Julbernardia globiflora* (Benth.) Troupin	Detarioideae	Miombo	X	X	X	X	X	X	X	NE	Tree 5–15 m
*Julbernardia paniculata* (Benth.) Troupin	Detarioideae	Miombo	X	X	X	X	X	X	X	NE	Tree 2–20 m
*Millettia stuhlmannii* Taub.	Papilionoideae	Miombo				X	X		X	NE	Tree 6–25 m
*Millettia usaramensis* Taub.	Papilionoideae	Miombo				X	X	X	X	NE	Shrub or tree 2–10 m
*Mundulea sericea* (Willd.) A. Chev.	Papilionoideae	Miombo	X		X	X	X	X	X	NE	Large shrub or tree 1–8 m
*Ormocarpum trichocarpum* (Taub.) Engl.	Papilionoideae	Miombo and Mopane				X	X	X	X	NE	Shrub or small tree 1–6 m
*Peltophorum africanum* Sond.	Caesalpinioideae	Miombo and Mopane	X	X	X	X		X	X	NE	Tree 3–12 m
*Pericopsis angolensis* (Baker) Meeuwen	Papilionoideae	Miombo	X	X	X	X	X	X	X	NE	Tree 3–20 m
*Philenoptera bussei* (Harms) Shrire	Papilionoideae	Miombo			X	X	X	X	X	NE	Tree 3–15 m
*Philenoptera nelsii* (Schinz) Schrire	Papilionoideae	Mopane	X		X	X				NE	Shrub or tree 4–12 m
*Philenoptera violacea* (Klotze) Shrire	Papilionoideae	Miombo	X	X	X	X	X	X	X	NE	Tree 4.5–27 m
*Pterocarpus angolensis* DC.	Papilionoideae	Miombo	X	X	X	X	X	X	X	LR/NT	Tree 5–30 m
*Pterocarpus brenanii* Barbosa & Torre	Papilionoideae	Miombo	X	X	X	X	X	X	X	LC	Tree 4–12 m
*Pterocarpus lucens* Guill. & Perr.	Papilionoideae	Miombo and Mopane	X		X	X		X	X	LC	Tree 7.5–18 m
*Pterocarpus rotundifolius* (Sond.) Druce	Papilionoideae	Miombo and Mopane	X	X	X	X	X	X	X	NE	Tree 3–25 m
*Senna petersiana* (Bolle) Lock	Caesalpinioideae	Miombo		X	X	X	X	X	X	NE	Shrub or small tree 2–6 m
*Tamarindus indica L.	Detarioideae	Miombo	X	X	X	X	X	X	X	LC	Tree to 25 m
*Xanthocercis zambesiaca* (Baker) Dumaz-le-Grand	Papilionoideae	Miombo			X	X		X	X	NE	Tree 7–30 m
*Xeroderris stuhlmannii* (Taub.) Mendonça & E.P. Sousa	Papilionoideae	Miombo and Mopane			X	X	X	X	X	NE	Tree up to 20 m
***Total numbers***	-										
**Number of species (Miombo)**	-	-	58	50	67	62	64	60	62	-	-
**Number of species (Mopane)**	-	-	19	11	21	23	17	18	17	-	-
**Number of species (Miombo and/or Mopane)**	-	-	62	51	71	66	65	62	64	-	-

* The origin of *Tamarindus indica* is uncertain and some sources (Africa Plant Database) suggest that it is native only in Madagascar. ^1^ Distribution: AO—Angola; DRC—Democratic Republic of Congo; ZM—Zambia; ZW—Zimbabwe; TZ—United Republic of Tanzania; MW—Malawi; MZ—Mozambique. ^2^ Conservation status according to IUCN Red List: LC—least concern; NT—near threatened; VU—vulnerable; NE—not evaluated.

**Table 2:** Leguminosae tree species from Miombo and Mopane ecosystems used in the phylogenetic analyses. GenBank accession numbers for the corresponding internal transcribed spacer (ITS) and cpDNA (rbcL and matK) sequences.

Taxon	Subfamily	rbcL	matK	ITS
*Acacia arenaria* Schinz	Caesalpinioideae	JX572181	JX517408	-
*Acacia erubescens* Welw. ex Oliv.	Caesalpinioideae	JF265248	JF270605	JQ265878
*Acacia galpinii* Burtt Davy	Caesalpinioideae	JX572194	JX518092	JQ265866
*Acacia gerrardii* Benth.	Caesalpinioideae	JF265250	JF270607	JQ265879
*Acacia hebeclada* DC.	Caesalpinioideae	JX572199	JX517672	JQ265920
*Acacia kirkii* Oliv.	Caesalpinioideae	JX572204	JX517387	JQ265829
*Acacia mellifera* (Vahl) Benth.	Caesalpinioideae	JX572211	JX517310	-
*Acacia nigrescens* Oliv.	Caesalpinioideae	EU213440	EU214210	KY688811
*Acacia nilotica* (L.) Willd. ex Delile	Caesalpinioideae	JF265255	JF270612	JX139101
*Acacia polyacantha*	Caesalpinioideae	-	-	JQ265902
*Acacia robusta* Burch.	Caesalpinioideae	JX572222	JX517547	-
*Acacia sieberiana* DC.	Caesalpinioideae	JF265259	JX517353	JQ265854
*Acacia welwitschii* Oliv.	Caesalpinioideae	JX572234	JX518159	-
*Afzelia quanzensis* Welw.	Detarioideae	JX572247	JX518045	KY306488
*Albizia adianthifolia* (Schum.) W.Wight	Caesalpinioideae	JQ025020	JQ024935	-
*Albizia anthelmintica* Brongn.	Caesalpinioideae	JX572254	JX517977	-
*Albizia antunesiana* Harms	Caesalpinioideae	-	-	-
*Albizia glaberrima* (Schum. & Thonn.) Benth.	Caesalpinioideae	JX572256	JX518104	-
*Albizia harveyi* E.Fourn.	Caesalpinioideae	JX572257	JX518176	-
*Albizia versicolor* Oliv.	Caesalpinioideae	JX572260	AF274210	-
*Amblygonocarpus andongensis* (Oliv.) Exell & Torre	Caesalpinioideae	JX572301	AF521812	-
*Baikiaea plurijuga* Harms	Detarioideae	JX572322	JX517704	KY306501
*Baphia bequaertii* De Wild.	Papilionoideae	-	-	-
*Baphia massaiensis* Taub.	Papilionoideae	JF265298	JF270652	-
*Bauhinia petersiana* Bolle	Cercidoideae	JX572327	JX517937	-
*Bauhinia thonningii* Schum.	Cercidoideae	KU568124	KT461985	-
*Bauhinia tomentosa* L.	Cercidoideae	JX572328	JX517621	KX057838
*Bobgunnia madagascariensis* (Desv.) J.H.Kirkbr. & Wiersema	Papilionoideae	JX572335	AY386940	EF560800
*Brachystegia allenii* Hutch. & Burtt Davy	Detarioideae	KU568100	KX146320	-
*Brachystegia bakeriana* Burtt Davy & Hutch.	Detarioideae	-	-	-
*Brachystegia boehmii* Taub	Detarioideae	JX572347	EU361886	KY306513
*Brachystegia floribunda* Benth.	Detarioideae	KU568148	KX146363	KY306515
*Brachystegia gossweileri* Burtt Davy & Hutch.	Detarioideae	-	-	-
*Brachystegia longifolia* Benth.	Detarioideae	KU568078	KX146300	AF513687
*Brachystegia puberula* Burtt Davy & Hutch.	Detarioideae	-	-	-
*Brachystegia spiciformis* Benth	Detarioideae	-	EU361888	KY306518
*Brachystegia tamarindoides* Benth.	Detarioideae	-	-	-
*Brachystegia taxifolia* Harms	Detarioideae	-	-	-
*Brachystegia utilis* Hutch. & Burtt Davy	Detarioideae	-	-	-
*Brachystegia wangermeeana* De Wild.	Detarioideae	-	-	-
*Burkea africana* Hook.	Caesalpinioideae	JQ025025	JQ024940	KX057840
*Cassia abbreviata* Oliv.	Caesalpinioideae	JX572384	JF270682	-
*Colophospermum mopane* (Benth.) Leonard	Detarioideae	JF265343	JF270696	AY955788
*Cordyla africana* Lour.	Papilionoideae	JF265371	KP177913	-
*Craibia zimmermannii* (Harms) Dunn	Papilionoideae	JX572478	JX518072	-
*Dalbergia arbutifolia* Baker	Papilionoideae	JX572499	JX517956	AB828608
*Dalbergia boehmii* Taub.	Papilionoideae	JX572501	JX517962	AB828617
*Dalbergia melanoxylon* Guill. & Perr.	Papilionoideae	KU748232	KY484235	KM276150
*Dalbergia nitidula* Baker	Papilionoideae	-	JX970899	-
*Dialium englerianum* Henriq.	Dialioideae	-	-	-
*Dichrostachys cinerea* (L.) Wight & Arn.	Caesalpinioideae	JQ025041	KX302328	AF458820
*Elephantorrhiza goetzei* (Harms) Harms	Caesalpinioideae	JX572549	JX517358	-
*Entada abyssinica* Steud. ex A. Rich.	Caesalpinioideae	JX572556	AF521829	KX057869
*Erythrophleum africanum* (Benth.) Harms	Caesalpinioideae	JX572568	JX517525	-
*Faidherbia albida* (Delile) A.Chev.	Caesalpinioideae	KX119293	KX119382	KX057872
*Guibourtia coleosperma* (Benth.) J. Léonard	Detarioideae	JX572650	JX518076	-
*Isoberlinia angolensis* (Benth.) Hoyle & Brenan	Detarioideae	KU568126	KX146343	HM041837
*Isoberlinia scheffleri* (Harms) Greenway	Detarioideae	AM234240	EU361983	HM041838
*Isoberlinia tomentosa* (Harms) Craib & Stapf	Detarioideae	KX119306	KX162205	KX057885
*Julbernardia globiflora* (Benth.) Troupin	Detarioideae	JX572701	JX517829	-
*Julbernardia paniculata* (Benth.) Troupin	Detarioideae	KU568145	KX146360	-
*Millettia stuhlmannii* Taub.	Papilionoideae	JX572773	JX517411	-
*Millettia usaramensis* Taub.	Papilionoideae	JX905971	JX905956	-
*Mundulea sericea* (Willd.) A. Chev.	Papilionoideae	JQ025063	JQ024975	AF467482
*Ormocarpum trichocarpum* (Taub.) Engl.	Papilionoideae	JX572810	JX517885	AF068158
*Peltophorum africanum* Sond.	Caesalpinioideae	JX572846	KX302342	-
*Pericopsis angolensis* (Baker) Meeuwen	Papilionoideae	KU568030	KX584412	KX584402
*Philenoptera bussei* (Harms) Shrire	Papilionoideae	JX572848	JX518116	-
*Philenoptera nelsii* (Schinz) Schrire	Papilionoideae	-	-	-
*Philenoptera violacea* (Klotze) Shrire	Papilionoideae	JF265547	JF270890	JX506439
*Pterocarpus angolensis* DC.	Papilionoideae	KY829237	KY829168	KY829139
*Pterocarpus brenanii* Barbosa & Torre	Papilionoideae	JX572903	JN083540	JN083475
*Pterocarpus lucens* Guill. & Perr.	Papilionoideae	KU568062	KX146285	JN083486
*Pterocarpus rotundifolius* (Sond.) Druce	Papilionoideae	JF265565	JF270907	JN083509
*Senna petersiana* (Bolle) Lock	Caesalpinioideae	JF265596	JX517765	-
*Tamarindus indica* L.	Detarioideae	AB378732	JQ587877	KY306654
*Xanthocercis zambesiaca* (Baker) Dumaz-le-Grand	Papilionoideae	JX573092	JX517427	-
*Xeroderris stuhlmannii* (Taub.) Mendonça & E.P. Sousa	Papilionoideae	JX573093	JX517470	AF467485

## Supplementary Materials

The following are available online at https://www.mdpi.com/2223-7747/8/6/182/s1, Figure S1: The phylogenetic trees obtained using the matK matrix with ML (A) and BI (B). Figure S2: The phylogenetic trees obtained using the rcbL matrix with ML(A) and BI (B). Figure S3: The phylogenetic trees obtained using the ITS matrix with ML (A) and BI (B). Table S1: Summary of the analyzed molecular data.
