# Supplementary material for: Diversification of African Tree Legumes in Miombo–Mopane Woodlands
Source: Plants (Basel). 2019 Jun 20;8(6):182. doi: 10.3390/plants8060182 (PMC6631767; doi:10.3390/plants8060182)
Supplement: Supplementary file 1 [file plants-08-00182-s001.pdf]

Figure S1: The phylogenetic trees obtained using the matK matrix with ML (A) and BI (B)

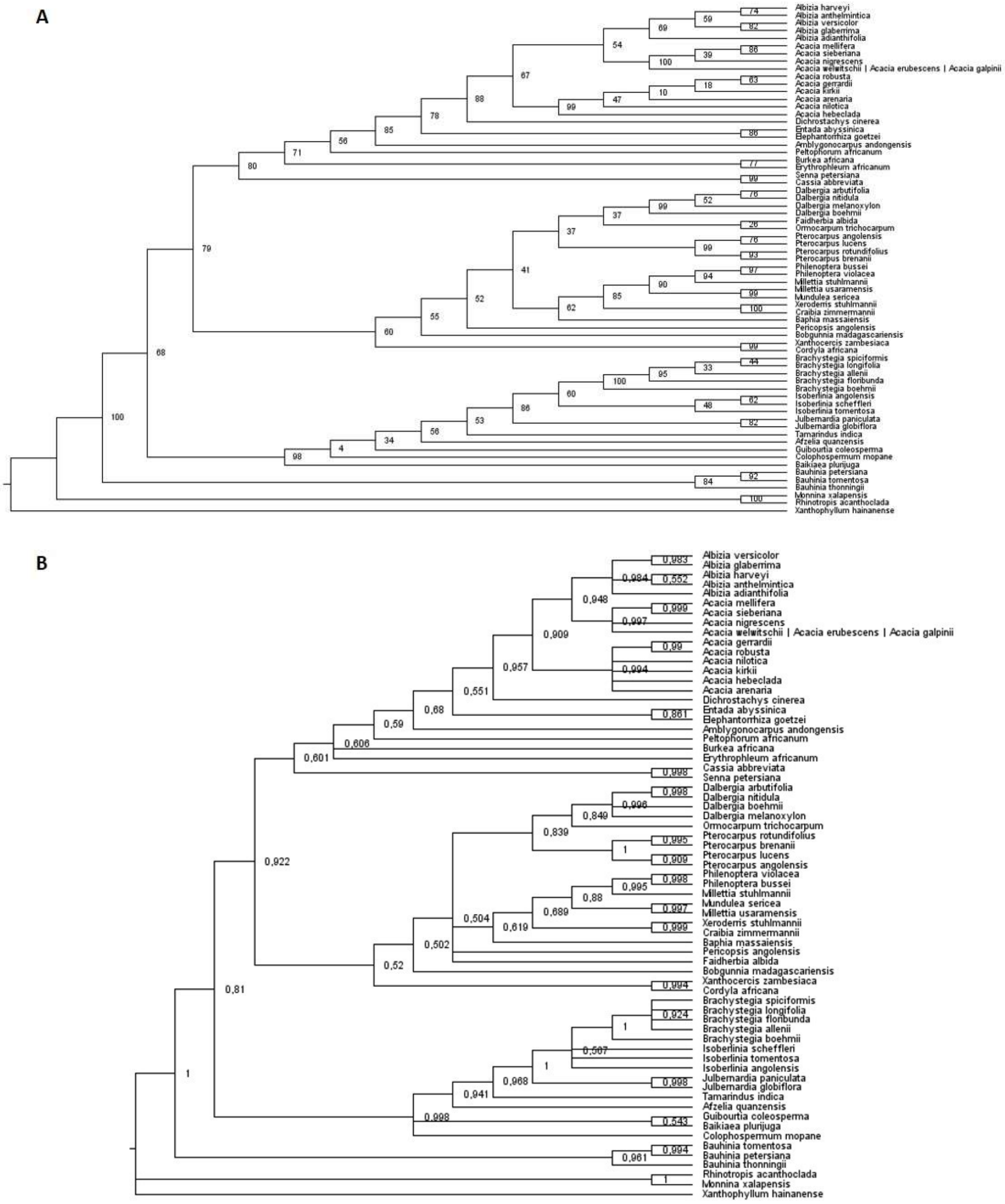

Figure S2: The phylogenetic trees obtained using the rcbL matrix with ML(A) and BI (B)

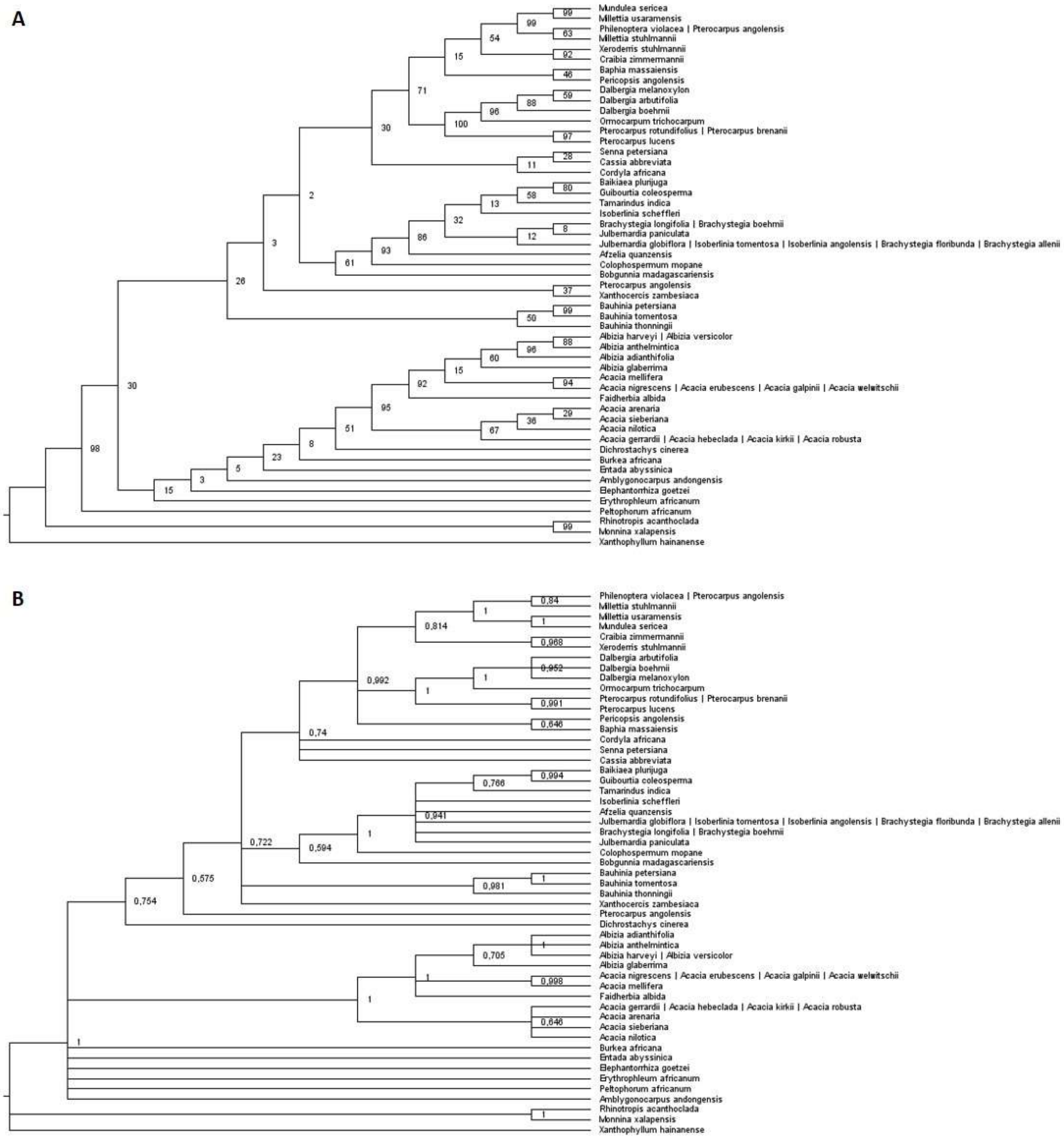

Figure S3: The phylogenetic trees obtained using the ITS matrix with ML (A) and BI (B).

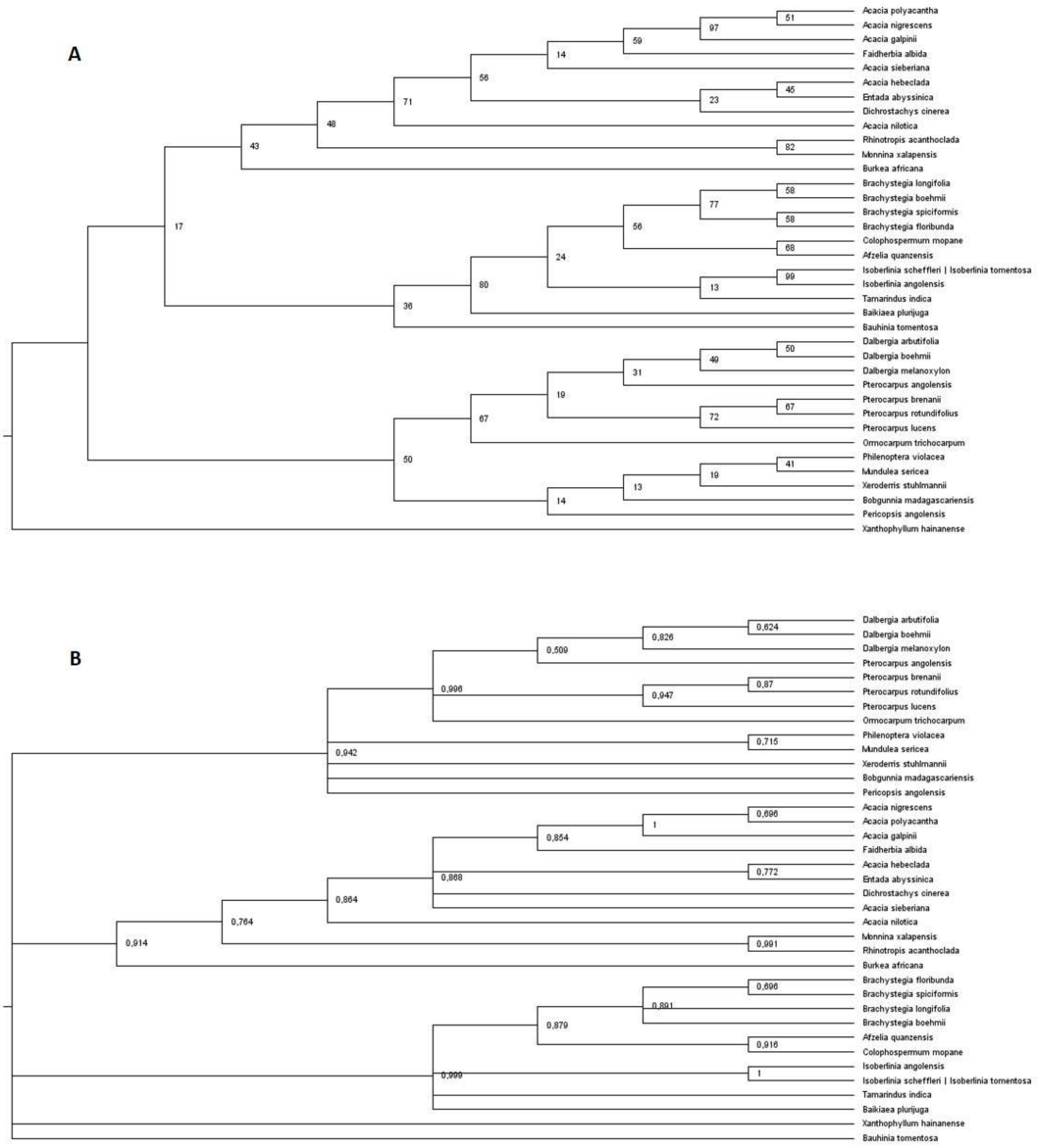

Table S1. Summary of the analysed molecular data.

	ITS	matK	rbcL	Combined
**No. Sequences**	41	69	67	70
**No. Haplotypes**	37	67	53	69
**No. Miombo species**	35	63	61	64
**No. Mopane species**	16	22	22	22
**No. Miombo and Mopane species**	12	19	19	19
